# Psychological Literacy Weakly Differentiates Students by Discipline and Year of Enrolment

**DOI:** 10.3389/fpsyg.2016.00162

**Published:** 2016-02-16

**Authors:** Brody Heritage, Lynne D. Roberts, Natalie Gasson

**Affiliations:** ^1^School of Psychology and Exercise Science, Murdoch University, PerthWA, Australia; ^2^School of Psychology and Speech Pathology, Curtin University, PerthWA, Australia

**Keywords:** psychological literacy, known-groups validity, measurement, undergraduate psychology, graduate attributes

## Abstract

Psychological literacy, a construct developed to reflect the types of skills graduates of a psychology degree should possess and be capable of demonstrating, has recently been scrutinized in terms of its measurement adequacy. The recent development of a multi-item measure encompassing the facets of psychological literacy has provided the potential for improved validity in measuring the construct. We investigated the known-groups validity of this multi-item measure of psychological literacy to examine whether psychological literacy could predict (a) students’ course of enrolment and (b) students’ year of enrolment. Five hundred and fifteen undergraduate psychology students, 87 psychology/human resource management students, and 83 speech pathology students provided data. In the first year cohort, the reflective processes (RPs) factor significantly predicted psychology and psychology/human resource management course enrolment, although no facets significantly differentiated between psychology and speech pathology enrolment. Within the second year cohort, generic graduate attributes (GGAs) and RPs differentiated psychology and speech pathology course enrolment. GGAs differentiated first-year and second-year psychology students, with second-year students more likely to have higher scores on this factor. Due to weak support for known-groups validity, further measurement refinements are recommended to improve the construct’s utility.

## Introduction

The construct of psychological literacy has become an integral part of discussions around the skills a graduate from a psychology degree should have (McGovern et al., 2010; Cranney et al., 2011b, 2012; Trapp et al., 2011; Mair et al., 2013; Karantzas, 2014; Baker, 2015). Psychological literacy is most commonly defined as “…the general capacity to adaptively and intentionally apply psychology to meet personal, professional, and societal needs" (Cranney et al., 2012, p. iii). It is theorized to consist of nine facets: psychological knowledge, scientific thinking, critical thinking, application of psychological principles, ethical behavior, information literacy, effective communication competence, respect for diversity and insight (McGovern et al., 2010). Current research is focusing on how to operationally define and subsequently measure psychological literacy (Cranney et al., 2011a; Karantzas, 2014; Roberts et al., 2015). However, there remain some questions as to whether psychological literacy should be seen as a desirable goal for university graduates from all disciplines, or whether psychological literacy should be seen as the primary goal of a psychology education and as a set of skills that sets the psychology graduate apart from other health professions.

**FIGURE 1:**
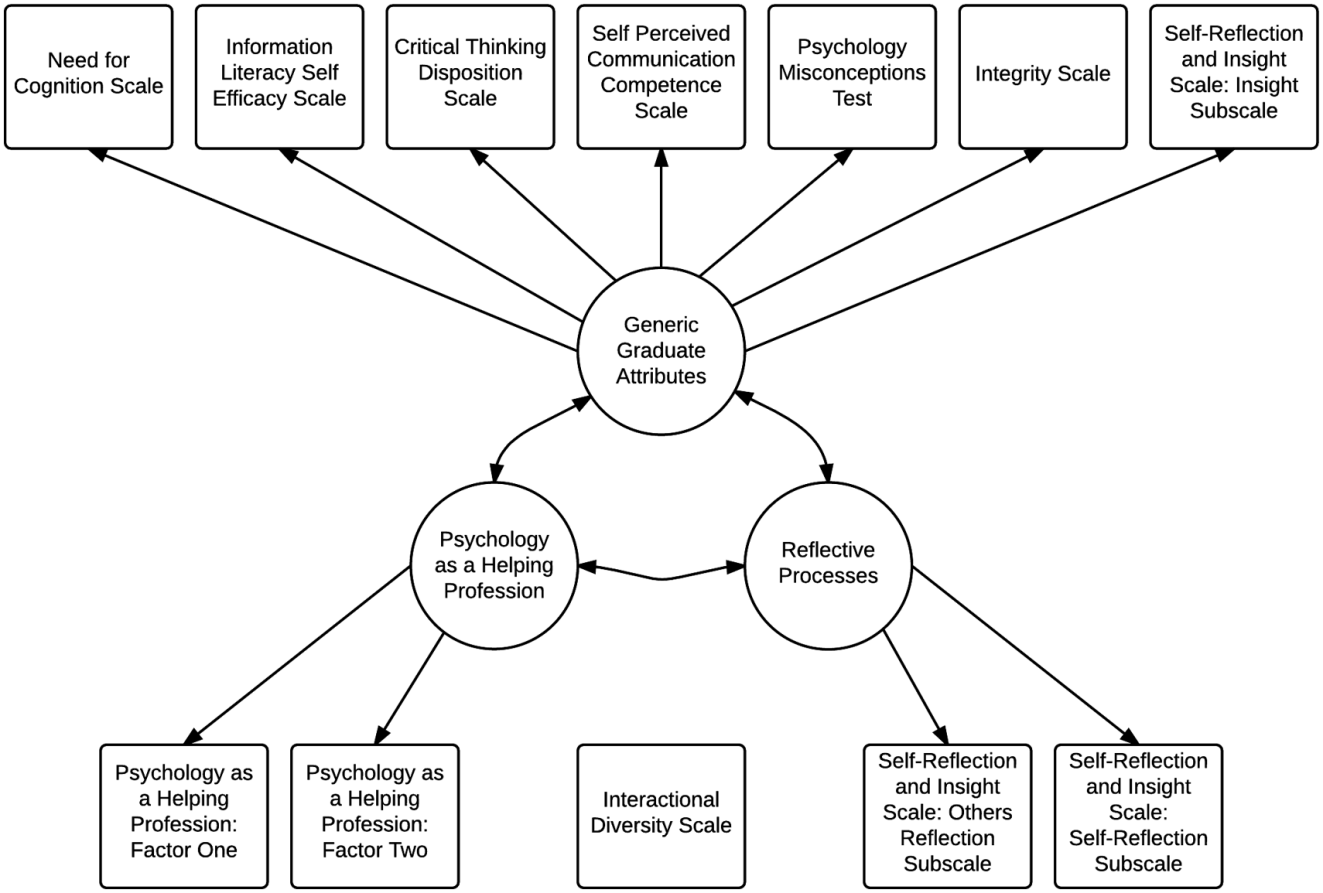
**Indicator measures and latent factors of psychological literacy, adapted from Roberts et al. (2015)**.

Our previous research (Roberts et al., 2015) examined the factor structure of self-report measures of the nine facets of psychological literacy defined by McGovern et al. (2010) and found three underlying factors and one independent construct (interactional diversity). The three factors were reflective processes (RPs), generic graduate attributes (GGAs), and psychology as a helping profession (PHP) (see **Figure 1**). The RPs factor comprised self- and other-reflection. The GGAs factor comprised scientific thinking, information literacy, communication competence, ethical behavior, insight, and critical thinking. Critical thinking loaded on RPs in one sample, and on GGAs in a second sample, with the latter loading argued by the authors as being more valid due to the distinction between reflecting on the behavioral or mental processes of the self and others (RPs), and the applied problem-solving focus of the critical thinking items (a generic university graduate attribute). PHP comprised personal growth and applied helping. The finding of both generic and psychology-specific factors in our previous research (Roberts et al., 2015) suggested that only some aspects of psychological literacy may be specific to psychology graduates, but this has yet to be tested.

This paper builds on the findings of our previous research (Roberts et al., 2015) by further examining the validity of the self-report measures of psychological literacy. In particular, we focus on known-groups validity. This type of validity is based on the proposition that for a test to be valid it must be able to discriminate between groups that theoretically differ (Hattie and Cooksey, 1984).

Psychological literacy is a skill taught as part of a psychology degree, and differences in psychological literacy between students from psychology and non-psychology courses would be expected using valid tests. Differences between disciplines in terms of what students are taught about psychological literacy have been identified. Murdoch et al. (2014) argue that there are three characteristics that set graduates of psychology apart from other health professionals. These are (1) knowledge and use of the scientific method of enquiry, (2) psychological literacy, and (3) the combined skills and knowledge of case formulation and diagnosis. Murdoch et al. (2014) reviewed the amount and type of mental health training provided to students of psychology, nursing, social work, and medicine across universities in Canada. The data showed that psychology students received more instruction in psychological literacy in comparison to nursing, social work, and medical students, with the latter disciplines receiving approximately the same amount of instruction in psychological literacy as each other. If the self-report measures of psychological literacy are valid, they should be able to discriminate between psychology students and students from other disciplines.

It is expected that psychology and non-psychology students would vary in terms of their capabilities in domains relevant to psychological literacy even at the onset of their enrolment based on selection effects. Schneider’s (1987) Attraction Selection Attrition theory contends that individuals are drawn to organizations (and by extension, university courses) on the basis of perceived similarities. Supporting this, Boone et al. (2004) have demonstrated in a tertiary education context that students self-select into courses based on personality similarities. These findings are consistent with Holland’s (1996) extensive work on the similarities of individuals within particular occupations, and its extension in the context of tertiary education, demonstrating that students tend to perceive fit with their course based on individual differences factors such as interests and personality. We may therefore anticipate that students self-selecting into psychology degrees, based on future careers in areas where psychological literacy is valuable and congruent, may at the onset of enrolment into a psychology degree have higher levels of psychological literacy than peers enrolling in other disciplines. Valid measures of psychological literacy factors should be able to discriminate between students entering psychology degrees and students entering other disciplines.

Furthermore, differences in psychology literacy at the time of enrolment are likely to continue to manifest beyond the first year of study for two reasons. First, as noted above, psychology students receive more education in psychological literacy than students enrolled in non-psychology courses. Second, Schneider’s (1987) Attraction Selection Attrition theory suggests that psychology students who perceive discrepancies between themselves and their course (and, consequently, the facets of psychological literacy represented within their coursework) are less likely to continue their education in this field. This homogenizing process of the individual differences for students enrolled in psychology and non-psychology courses progress would therefore consolidate expected differences in psychological literacy between courses beyond the onset of enrolment. Psychological literacy measures should therefore discriminate between students enrolled in psychology majors and students enrolled in other majors. One study provides preliminary support for this hypothesis. Morris et al. (2013) examined awareness, importance, and perceived development of psychological literacy in 213 students taking undergraduate psychology units. Students were grouped into non-psychology majors (students taking a psychology unit[s] as an elective), psychology majors (students enrolled in either of the two accredited undergraduate psychology degrees) and experienced psychology majors (students enrolled in one of two accredited psychology degrees who had also completed the course capstone unit). While there were no significant effects of group or year level for the *importance* placed on psychological literacy there was a significant group effect for how much students thought their psychological literacy had *developed* during their studies. Experienced majors reported more development than majors, and majors reported more development than non-majors.

If psychological literacy develops as a function of undergraduate education in psychology, we might also expect psychological literacy to increase with years of psychology education. Morris et al.’s (2013) study also provided preliminary support for this hypothesis. There was a significant increase in the reported development of psychological literacy across the years of the psychology degree. In addition, there were significant correlations between the number of psychology units a student had completed and the importance and development ratings they gave. However, it must be noted that a limitation of this study is that the relative importance and development of psychological literacy were measured using single item measures of unknown reliability or validity. Valid measures of psychological literacy factors should be able to discriminate between students of differing years of study.

## The Current Study

In summary, our previous research (Roberts et al., 2015) began the process of measuring the nine proposed facets of psychological literacy in undergraduate psychology students using single and multi-item self-report measures. This research focused on determining the factor structure underlying psychological literacy, finding generic and psychology-specific factors. In the current study, we build on this research to examine the known-groups’ validity of the psychological literacy self-report measures. If students entering university are attracted to study particular disciplines based on pre-existing interests and personality traits we might also expect that students entering a psychology degree will already be higher on the psychology-specific factors of psychological literacy than students entering other disciplines (H1). If psychological literacy is something that is taught in undergraduate psychology degrees, we would expect that the psychology-specific factors of psychological literacy would discriminate between psychology students and students from other disciplines beyond the first year (H2). Similarly, we would expect that students who have completed more psychology education would score higher on psychology specific factors of psychological literacy than those who have completed less psychology education. That is, psychological literacy variables would significantly differentiate students from each year of study (H3).

## Materials and Methods

### Participants

The participants for this research were a convenience sample of 886 students at an Australian university. Of this sample, 74 participants did not report their discipline of study (*N* = 43), or were enrolled in a course that was not common in our sample thereby enhancing the risk of overdispersion during later analysis (*N* = 31) (Tabachnick and Fidell, 2013). These cases were subsequently removed from any further analysis. Ninety-nine cases where participants identified English as not being their first language were removed from further analyses due to the potentially biased data. Participants enrolled in their fourth year, honors, or postgraduate programs of study were not represented in sufficient quantity for each reported discipline to meet the frequency assumptions of the forthcoming logistic regression analyses, and were omitted from further analysis (*N* = 28). Of the remaining 685 participants, 515 were enrolled in a psychology degree, 87 were enrolled in a psychology and human resource management double-degree, and 83 were enrolled in a speech pathology degree^1^. **Table 1** presents a summary of the demographic variables measured by the course of enrolment. Part of this sample (students enrolled in a psychology course) has previously been used in Roberts et al. (2015) to assess the factor structure of the multi-item facet measures of psychological literacy.

**Table 1:** Demographic characteristics of the undergraduate participants (*N* = 685).

	**Psychology**	**Psychology-HRM**	**Speech pathology**
Total *N*	515	87	83
**Age ^a^**			
*M (SD)*	21.01 (5.89)	19.72 (3.70)	21.91 (6.77)
**Gender *N***			
Male	121	15	3
Female	394	72	80
**Year of study in current course *N***			
First year undergraduate	210	37	17
Second year undergraduate	275	36	66
Third year undergraduate	30	14	0
**Years of university completed ^b^**			
*M (SD)*	1.50 (1.65)	1.43 (1.44)	1.55 (1.20)
**Psychology classes in highschool *N***			
Yes	95	15	15
No	420	72	68
**Enrolment mode *N***			
Full-time	435	78	66
Part-time	78	9	17
**Enrolment origin *N***			
Domestic	503	87	79
International	12	0	4

*Some demographic items had marginal missing data (*<*1%).*^a^*Measured in years.*^b^*Years of university completed includes those related to the student’s currently enrolled course and any prior university studies they have completed.*

### Measures

An online questionnaire was developed comprising single-item and multi-item measures of the nine facets of psychological literacy, and demographic items (age, gender, years of study, the number of psychology units completed, full-time or part-time status, international, or domestic student). Only the multi-item measures of psychological literacy were used in the current study. A summary of the measures is presented in **Table 2.** Further details of the measures, including their psychometric properties, are provided in Roberts et al. (2015).

**Table 2:** Measures of psychological literacy facets.

Construct	Measure	Reference	Example item	No. Items	Latent Factor
Psychological knowledge	Psychology misperceptions test	Hughes et al., 2013	“People predominantly use either the left side or the right side of their brain”	29	GGA
Scientific thinking	Need for cognition	Cacioppo et al., 1984	“I find satisfaction in deliberating hard and for long hours”	18	GGA
Critical thinking	Critical thinking disposition scale	Sosu, 2013	“I usually check the credibility of the source of information before making judgments”	11	GGA
Application to issues	Psychology as a helping profession scale	Gervasio et al., 2010	“People can learn to enhance their health (e.g., stop smoking) through courses in psychology”	11	PHP
Acting ethically	The integrity scale	Schlenker, 2008	“It is important to me to feel that I have not compromised my principles”	18	GGA
Using/evaluation information and technology	Information literacy self-efficacy scale	Kurbanoglu et al., 2006	“Locate resources in the library using the library catalog”	17	GGA
Communicating effectively	Self-perceived communication competence scales	McCroskey and McCroskey, 1988	“Present a talk to a group of strangers”	12	GGA
Respect for diversity	Interactional diversity scale	Hu and Kuh, 2003	“Had serious discussions with students whose religious beliefs were very different from yours”	7	None
Self/other insight/reflection	Self-reflection and insight scale	Grant et al., 2002	“I frequently examine my feelings”	26	RP/GGA
Single item psychological literacy measures	–	Chester et al., 2013	“At this point in your education, how would you rate your knowledge of basic concepts/principles in Psychology?”	9	Not applicable

*GGA, generic graduate attributes; PHP, psychology as a helping profession; RP, reflective processes.*

### Procedure

Recruitment for the study was conducted in two time periods; the first semester of 2013 and 2014; following Curtin University Human Research Ethics Committee approval. Students were recruited through an announcement in psychology lectures and learning management system sites and through a school-based research participation pool. Students participating through the pool were awarded research points and all other participants were entered into a prize draw for a $100 Amazon.com voucher.

In line with best practize recommendations (Allen and Roberts, 2010), the online questionnaire was ‘sandwiched’ between a participant information sheet and a debriefing page hosted on the university website. Links were provided to the participant information sheet. Upon reading the participation information sheet and consenting to participate, students were redirected to the questionnaire. Survey data were downloaded from Qualtrics.com into SPSS (v. 20) for analysis. The data was screened for missing values and multiple responding. Due to the possible confound of the year of enrolment, data was split into first-year and second-year students for subsequent analyses regarding discipline differences in psychological literacy factors.

## Results

Regression scores for each factor of psychological literacy reflective of the model identified in Roberts et al. (2015) [see **Figure 1**] were calculated for each participant via confirmatory factor analytic methods. Correlations and variances of the psychological literacy factor scores are presented in **Table 3** and the factor scores were used in the forthcoming regression analyses.

**Table 3:** Bivariate correlations, variances, and reliabilities of the psychological literacy factors (*N* = 685).

	Psychology only, years one to three			All measured disciplines (first years)			All measured disciplines (second years)		
	GGA	PHP	RP	GGA	PHP	RP	GGA	PHP	RP
GGA									
PHP	0.424*^∗∗∗^*			0.414*^∗∗∗^*			0.540*^∗∗∗^*		
RP	0.854*^∗∗∗^*	0.448*^∗∗∗^*		0.755*^∗∗∗^*	0.365*^∗∗∗^*		0.879*^∗∗∗^*	0.493*^∗∗∗^*	
Variance	0.088	0.462	0.162	0.082	0.332	0.186	0.081	0.236	0.153

*GGA, generic graduate attributes; PHP, psychology as a helping profession; RP, reflective processes.*

^∗∗∗^*p < 0.001.*

### Psychological Literacy at the Time of Entering Degree by Discipline (H1)

Our first set of analyses was designed to test the known-groups validity of students entering the first year of their undergraduate studies (H1).

Multinomial logistic regression was used to predict group membership (psychology majors, psychology-HRM majors, and speech pathology majors) of the first year cohort. The predictor variables were the measures of each facet of psychological literacy. GGAs, PHP, and RPs were entered in a single block. Psychology majors were chosen as the reference group, as this allowed theoretically meaningful contrasts between psychology majors and the other two groups of majors being examined. All statistical assumptions were met prior to conducting the main analysis.

The predictor-inclusive model was significantly different from the baseline model, χ^2^(6) = 13.12, *p* = 0.041, indicating that the set of psychological literacy factors was capable of distinguishing between psychology majors and speech pathology/psychology HRM majors. Non-significant Pearson (*p* = 0.551) and Deviance (*p* = 1.000) model fitting statistics indicated good model fit. Cox and Snell *R*^2^ = 0.048, and Nagelkerke *R*^2^ = 0.067, indicated small-to-moderate effect sizes for the model by Cohen’s (1988) conventions. Parameter estimates for the model are presented in **Table 4.** Comparisons between psychology and psychology-HRM students indicated that the RPs factor scores were capable of differentiating group membership between the students. Psychology students were significantly more likely to have a higher factor score on RPs compared to that of the Psychology-HRM students within the first year sample. Within the first year cohort, however, there were no significant predictors of group membership between the psychology and speech pathology students.

**Table 4:** Parameter estimates for psychological literacy factors differentiating enrolment groups.

				95% *CI*	
	*B (SE)*	*p*	Exp(*B*)	Lower	Upper
**First year students**					
Psychology-HRM					
Intercept	–1.81 (0.19)				
GGA	0.41 (1.02)	0.691	1.50	0.20	11.18
PHP	0.35 (0.37)	0.347	1.42	0.69	2.92
RP	–1.47 (0.65)	0.023^∗^	0.23	0.06	0.82
Speech pathology					
Intercept	–2.65 (0.28)				
GGA	0.76 (1.39)	0.586	2.134	0.14	32.60
PHP	–0.61 (0.47)	0.193	0.54	0.22	1.36
RP	–1.27 (0.88)	0.151	0.28	0.05	1.58
**Second year students**					
Psychology-HRM					
Intercept	–2.12 (0.19)				
GGA	–0.16 (1.34)	0.907	0.86	0.06	11.91
PHP	–0.85 (0.45)	0.056	0.43	0.18	1.02
RP	–0.55 (0.95)	0.567	0.58	0.09	3.75
Speech pathology					
Intercept	–1.55 (0.15)				
GGA	2.35 (1.11)	0.034^∗^	10.47	1.20	91.50
PHP	–0.05 (0.35)	0.882	0.95	0.48	1.89
RP	–3.00 (0.79)	0.000^∗∗∗^	0.05	0.01	0.23

*Psychology students were the reference category during analysis. GGA, generic graduate attributes; PHP, psychology as a helping profession; RP, reflective processes.*

^∗∗∗^*p < 0.001.*

^∗^*p < 0.05.*

### Psychological Literacy in Second-Year Undergraduate Students (H2)

Multinomial logistic regression, using the same set of psychological literacy predictors, was conducted on the second year data to examine whether the prediction of group membership for psychology, psychology-HRM, and speech pathology majors was evident. All assumptions were validated prior to testing, and all psychological literacy factors were entered in one predictor block. The predictor-inclusive model was significantly different from the baseline model, χ^2^(6) = 30.88, *p* < 0.001, indicating that the set of psychological literacy factors predicted discipline membership for the second year data. Both non-significant Pearson (*p* = 0.289) and Deviance (*p* = 1.000) statistics indicated good model fit. Cox and Snell *R*^2^ = 0.079, and Nagelkerke *R*^2^ = 0.101, indicated small-to-moderate effect sizes for the model by Cohen’s (1988) conventions and were comparatively larger than that of the first year data model. Comparisons between psychology majors and psychology-HRM/speech pathology majors were again conducted, with psychology majors being the reference group. **Table 4** demonstrates the parameter estimates for each discipline comparison. Speech pathology group membership was predicted by both GGAs and RPs. Students were more likely to be a speech pathology student if they had a higher GGAs factor score, or a lower RPs score, in comparison to psychology students.

### Psychological Literacy by Year of Enrolment (H3)

Our third set of analyses was designed to test whether psychological literacy increased with psychology education. Only data from participants identifying as psychology majors was included for analysis (*N* = 515).

All assumptions were met prior to using multinomial logistic regression to predict year group membership from the factors of psychological literacy. The first year undergraduate group was set as the reference group for the analysis. The predictor-inclusive model significantly predicted year group membership based on the factors of psychological literacy, χ^2^(6) = 13.70, *p* = 0.033. Effect size, as reported by Cox and Snell *R^2^* = 0.026, and Nagelkerke *R^2^* = 0.032, were both small by Cohen’s (1988) conventions. Both non-significant Pearson (*p* = 0.370) and Deviance (*p* = 1.000) statistics indicated good model fit. Comparisons between first-year psychology undergraduates and second-year psychology undergraduates indicated a significant difference in GGAs. Students with higher GGA scores were significantly more likely to be second-year psychology students. Conversely, comparisons between first- and third-year psychology undergraduates did not indicate any significant indicators of group membership. The parameter coefficients are reported in **Table 5.**

**Table 5:** Parameter estimates for psychological literacy factors differentiating year groups for psychology majors.

				95% CI	
	*B (SE)*	*p*	Exp(B)	Lower	Upper
**Second year students**					
Intercept	0.27 (0.09)				
GGA	1.31 (0.61)	0.031^∗^	3.70	1.13	12.10
PHP	–0.19 (0.15)	0.226	0.83	0.61	1.12
RP	–0.22 (0.45)	0.630	0.81	0.33	1.94
**Third year students**					
Intercept	–2.00 (0.21)				
GGA	1.66 (1.31)	0.205	5.28	0.40	69.13
PHP	0.16 (0.33)	0.625	1.18	0.61	2.25
RP	–0.16 (1.00)	0.870	0.85	0.12	6.04

*First year students were the reference category during analysis. GGA, generic graduate attributes; PHP, psychology as a helping profession; RP, reflective processes.*

^∗^*p < 0.05.*

## Discussion

The purpose of the current study was to investigate whether the previously identified factors of psychological literacy (Roberts et al., 2015) were capable of differentiating group membership, in terms of course of enrolment or year of enrolment, as a test of known-groups validity. We predicted that participant scores on the factors of psychological literacy would significantly contribute to the prediction of group membership between psychology and non-psychology undergraduate students. At the time of entering their degree, group membership differences were predicted only by the factor of RPs between the psychology and psychology-HRM students, with the remaining psychological literacy factors not indicating any significant value in predicting group membership. Likewise, there was no notable differentiation between psychology and speech pathology students in this first-year sample. These results provided weak, partial support for H1. In predicting group membership within the second year cohort, more factors of psychological literacy were capable of distinguishing between psychology and speech pathology students. Psychology and speech pathology membership were predicted by GGAs and RPs scores. No psychological literacy factors were significant in predicting group membership between psychology and psychology HRM students. These findings provide partial support for H2. Lastly, we investigated whether year-group membership in psychology students could be predicted by the factors of psychological literacy, which were presumed to improve as psychology undergraduate education progressed. Weak support was provided for H3, as the first and second year psychology students were differentiated by GGAs, with higher GGA scores being more likely for second-year psychology students. No psychological literacy factors were influential in differentiating first and third year psychology students, contrary to what was expected for H3. Effect sizes for all analyses were small-to-moderate in size, providing limited support for the known-groups validity of these measures of psychological literacy.

These findings differ from what was predicted based on prior research. The limited ability of psychological literacy factor scores to predict group membership for the first year students departs from predictions from Schneider’s (1987) Attraction-Selection–Attrition framework. While we proposed that students were more likely to be attracted to a course based on existing individual-level similarities, which in turn would allow group membership prediction based on heterogeneity across courses, this was not supported. By the second year of enrolment, students in the three majors have all been exposed to some psychology education as part of an interprofessional first year course requirement, but have also been exposed to discipline-specific education. The second year sample of students varied on more facets of psychological literacy than first year students, perhaps attributable to discipline-specific education encouraging more-informed perceptions of match or mismatch with their course and future profession. This may have resulted in greater homogeneity within the student cohort of each course due to attrition where mismatches were perceived, promoting greater group membership prediction within the second year sample. Our findings are similar to Morris et al. (2013), with psychology and non-psychology students demonstrating a degree of heterogeneity in factors of psychological literacy, although in our analyses this heterogeneity was not present between psychology and psychology-HRM students

Consistent with the findings of Morris et al. (2013), we demonstrated support for psychological literacy differentiating group membership between years of enrolment in a psychology degree. Our findings, while limited, demonstrated that GGAs differentiated between psychology students at the time of course entry, and psychology students in their second year. These findings must be considered in the context of the whole model however; there was no differentiating effect between first and third year students, which is unusual if psychological literacy is theorized to improve as a function of course tenure (Morris et al., 2013). Furthermore, GGAs was the only significant predictor of first- and second-year group membership for psychology students, and this facet of psychological literacy has been considered previously by the authors as skills that are not psychology-specific, but likely learned by university students as part of their undergraduate progression (Roberts et al., 2015). Our findings therefore provide weak support for the efficacy of psychological literacy being capable of differentiating psychology students at different stages of their course progression.

From a measurement perspective, the measures selected to capture McGovern et al.’s (2010) conceptualisation of psychological literacy may not be optimal, and this may be reflected in the limited support for known-groups validity in the current study. For example, Roberts et al. (2015) noted that the factor structure coefficients and model fit values for the three-factor model underlying psychological literacy could benefit from further improvement. The opportunity to examine the factor structure of the facets of psychological literacy for speech pathology and psychology-HRM double degree students was not tenable with the current sample due to the small sample sizes (Kline, 2010). Roberts et al. (2015) identified issues with low standardized factor loadings, indicating the need to further examine the indicators of latent factors of psychological literacy. This may in turn reduce the prospect of attenuated relationships between indicators and factors, thereby reducing the prospect of type II errors when examining the predictive validity of psychological literacy and other outcomes. Additionally, investigation of the construct validity of the three-factor model of psychological literacy by Roberts et al. (2015) based on samples from other universities, would provide valuable information on the model’s generalizability. While the exploratory nature of Roberts et al. (2015) and the current study provides a first step in examining the predictive and construct validity of psychological literacy, respectively, further work is needed.

Our recommendations for future research therefore fall into two broad categories: the further evaluation of the way in which psychological literacy is measured, and the examination of the three factor model of Roberts et al. (2015) with other samples. To address the first recommendation, we propose that future research investigating psychological literacy may benefit from trialing smaller subsets of items that aim to tap into the factors underlying the construct. While Roberts et al. (2015) and the current study have used existing measures that were considered to reflect each of the facets of psychological literacy proposed by McGovern et al. (2010), designing and trialing a parsimonious measure that reflects these facets would be advantageous. A reduction in the number of scale items would also be beneficial in terms of reducing respondent fatigue during administration.

Addressing the first recommendation may consequently provide evidence that addresses our second recommendation, which is the need for future research to further examine the validity of Roberts et al.’s (2015) three-factor model of psychological literacy. While the large psychology student samples from Roberts et al. (2015) provided a sufficiently powered analysis of the factor structure of psychological literacy, the need to test this model with samples from other universities and other disciplines is a valuable future direction. We examined whether the three factors of psychological literacy could predict group membership between courses that were based on health-focused interaction with other persons. Stronger results may be obtained when comparing courses from less related disciplines, including those that do not provide any psychology units in their undergraduate coursework. Courses such as engineering, which Holland’s (1996) interest-major typology suggests would attract and retain students with pronounced differences in personality/interests in comparison to health-sciences students, may demonstrate greater differences in psychological literacy. By addressing these current limitations, the construct of psychological literacy may be a valuable means of representing the skills developed during a psychology degree.

## Author Contributions

All authors listed, have made substantial, direct and intellectual contribution to the work, and approved it for publication.

## Conflict of Interest Statement

The authors declare that the research was conducted in the absence of any commercial or financial relationships that could be construed as a potential conflict of interest.

## Footnotes

1 We can be confident that psychology students in second year and beyond were not students who had newly switched into a psychology major, as second and third year units have prerequisites of the completion of psychology units from the previous year.
